# Role of Carnitine in Non-alcoholic Fatty Liver Disease and Other Related Diseases: An Update

**DOI:** 10.3389/fmed.2021.689042

**Published:** 2021-08-09

**Authors:** Na Li, Hui Zhao

**Affiliations:** ^1^Second Affiliated Hospital of Dalian Medical University, Dalian, China; ^2^Department of General Practice, Xi'an People's Hospital (Xi'an Fourth Hospital), Xi'an, China; ^3^Department of Health Examination Center, The Second Affiliated Hospital of Dalian Medical University, Dalian, China

**Keywords:** carnitine, non-alcoholic fatty liver disease, L-carnitine supplementation, targeted therapy, therapeutic diet

## Abstract

Carnitine is an amino acid-derived substance that coordinates a wide range of biological processes. Such functions include transport of long-chain fatty acids from the cytoplasm to the mitochondrial matrix, regulation of acetyl-CoA/CoA, control of inter-organellar acyl traffic, and protection against oxidative stress. Recent studies have found that carnitine plays an important role in several diseases, including non-alcoholic fatty liver disease (NAFLD). However, its effect is still controversial, and its mechanism is not clear. Herein, this review provides current knowledge on the biological functions of carnitine, the “multiple hit” impact of carnitine on the NAFLD progression, and the downstream mechanisms. Based on the “multiple hit” hypothesis, carnitine inhibits β-oxidation, improves mitochondrial dysfunction, and reduces insulin resistance to ameliorate NAFLD. L-carnitine may have therapeutic role in liver diseases including non-alcoholic steatohepatitis, cirrhosis, hepatocellular carcinoma, alcoholic fatty liver disease, and viral hepatitis. We also discuss the prospects of L-carnitine supplementation as a therapeutic strategy in NAFLD and related diseases, and the factors limiting its widespread use.

## Introduction

Carnitine (3-hydroxy-4-N-trimethylammoniobutanoate) is an essential water-soluble molecule with multiple functions in the human body (1). Examples of such functions include reducing oxidative stress, increasing expression of pro-inflammatory cytokines (2–4), and improving mitochondrial dysfunction (5) and insulin resistance (IR) (6, 7). Moreover, it plays an important role in the development of many metabolic diseases, such as hypertension, diabetes, polycystic ovary syndrome (8), and osteoarthritis (9). Besides, carnitine has been reported to be closely associated with the development of non-alcoholic fatty liver diseases (NAFLD). Numerous studies have shown that NAFLD has become a major healthcare concern and economic burden worldwide (10); therefore, its prevention and treatment have gained increased attention among researchers. In this review, we summarize and discuss the relationship between carnitine and NAFLD, effects of L-carnitine (the biologically active form of carnitine) supplementation in NAFLD, and related diseases, including non-alcoholic steatohepatitis (NASH), cirrhosis, hepatic cellular cancer (HCC), alcoholic fatty liver disease, and viral hepatitis.

## Biological Characteristics of Carnitine

### Classification and Distribution in Human Body

Carnitine is an amino acid belonging to a quaternary ammonium cationic complex. It has two stereoisomers: bioactive L-carnitine and abiotic enantiomeric isomer D-carnitine. L-carnitine is the predominant carnitine used in biological and medical fields and is commonly referred to as “carnitine.” The human body contains about 300 mg/kg of L-carnitine, 98% of which is intracellular, with 80% present in the muscles, 5–10% in the gastrointestinal tract, and 3% in the liver (11). Although D-carnitine has no biological activity in humans, it adversely impacts biochemical processes by inhibiting the carnitine acetyltransferase (12). D-carnitine supplementation has been found to induce liver inflammation, oxidative stress, and apoptosis in animal studies (13). In addition, D-carnitine can cause secondary carnitine deficiency (SCD), so researchers often use D-carnitine supplements to feed mice to establish carnitine lack animal models (14, 15). For these reasons, D-carnitine has been rarely studied in humans.

### Endogenous Synthesis and Exogenous Sources

**Figure 1:**
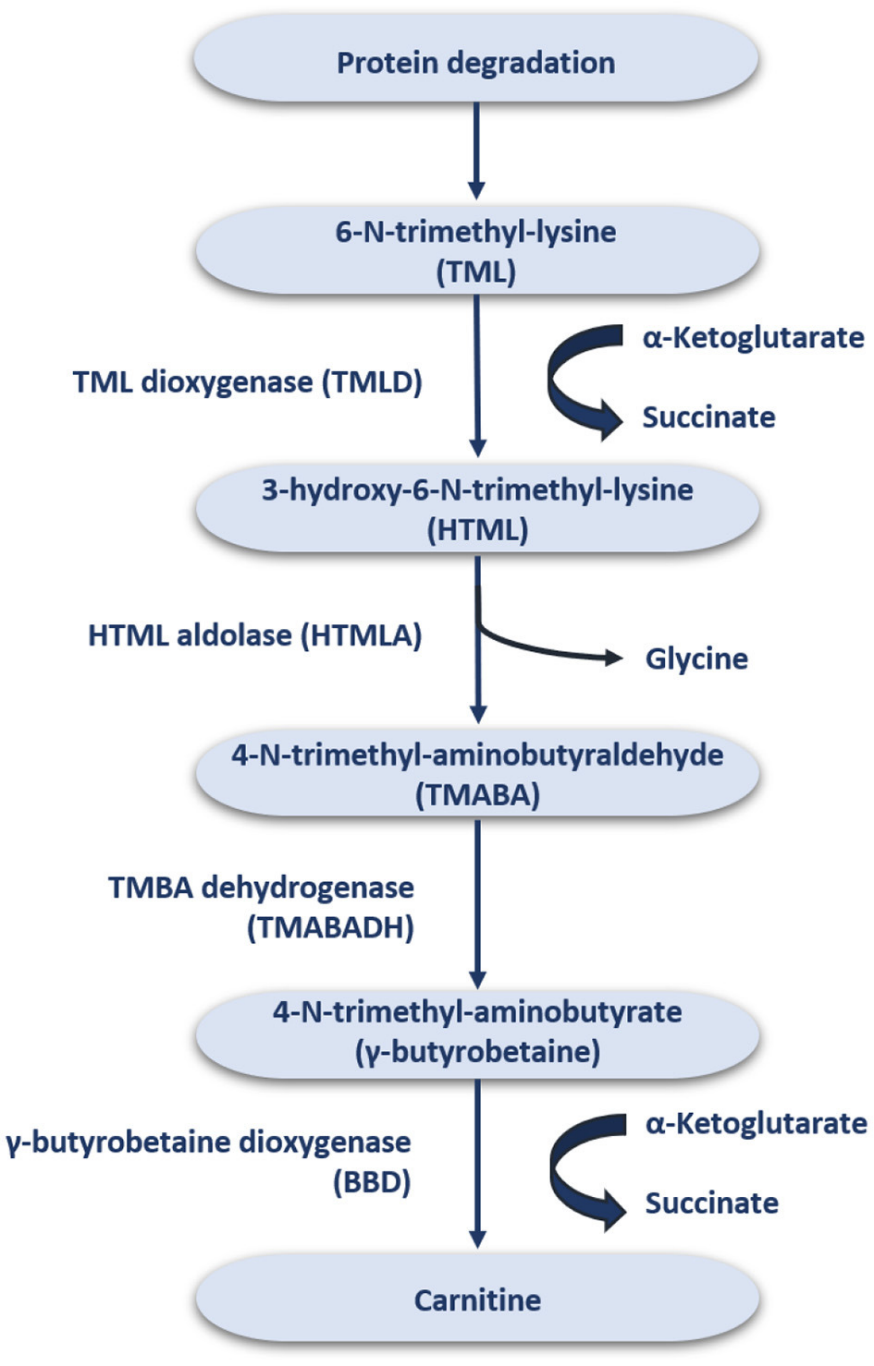
The endogenous synthesis of carnitine.

In the average adult diet, it is estimated that about 75% of the daily carnitine requirement mainly comes from meat, fish, and dairy products (16), while the remaining 25% is derived from endogenous synthesis (1). In strict vegetarians, more than 90% of the daily carnitine requirement is obtained by endogenous synthesis (17–19), whereby humans can synthesize ~1–2 μmol carnitine/kg/day (20). The synthesized carnitine is formed when 6-N-trimethyl-lysine (TML) is released during protein degradation (Figure 1) (21). After release, TML is hydroxylated into 3-hydroxyl-6-N-trimethyl-lysine (HTML) by trimethyl dioxygenase (TMLD), which is then broken down into 4-N-trimethyl-butylaldehyde (TMABA) and glycine by HTML aldolase (HTMLA) (1). TMABA produces 4-N-trimethylaminobutyrate (γ-butyrobetaine) under the action of dehydrogenase (11). Finally, γ-butyrobetaine dioxygenase (BBD) is used to hydroxylate γ-butyrobetaine to produce endogenous carnitine (Figure 1) (22, 23). BBD is confined to the human liver, kidneys, testis and brain, thus the biosynthesis of carnitine occurs only at these locations (24). Other tissues, such as skeletal muscle, obtain carnitine from the blood (25).

The distribution and homeostasis of carnitine within the body is controlled by organic cationic transporters (OCTN) (26). OCTN act on intestinal absorption and renal reabsorption of carnitine, and plays an important role in tissue distribution by catalyzing carnitine to enter cells *in vivo* (27). Among these, OCTN2 is the most important physiologically transporter of carnitine due to its high affinity and wide expression (27). OCTN2 plays a crucial role in carnitine homeostasis. Notably, BB, a direct precursor of carnitine, is also a good substrate for OCTN2. The liver and kidneys have a strong ability to convert BB to carnitine. Loss or mutation of OCTN2 function results in primary systemic carnitine deficiency (PCD) with severe clinical consequences such as cardiac and skeletal myopathy, cardiac hypertrophy and NAFLD (28).

### Biological Functions

#### Transport of Long-Chain Fatty Acids Into the Mitochondrial Matrix

The essential function of carnitine is to transport LCFAs from the cytoplasm to the mitochondrial matrix for subsequent degradation by β-oxidation, known as “carnitine shuttle” (5). LCFA activation occurs in the cytosol, but the enzymes required to catalyze LCFA oxidation exist in the mitochondrial matrix (29). In this process, LCFA must be first activated into lipoyl-CoA via acyl-CoA synthetase (ACS) (30). Then, lipoyl-CoA is transported into the mitochondria. Since the inner mitochondrial membrane is impermeable to lipoyl-CoA (29), the entry of lipoyl-CoA relies on a shuttle system, which requires carnitine.

**Figure 2:**
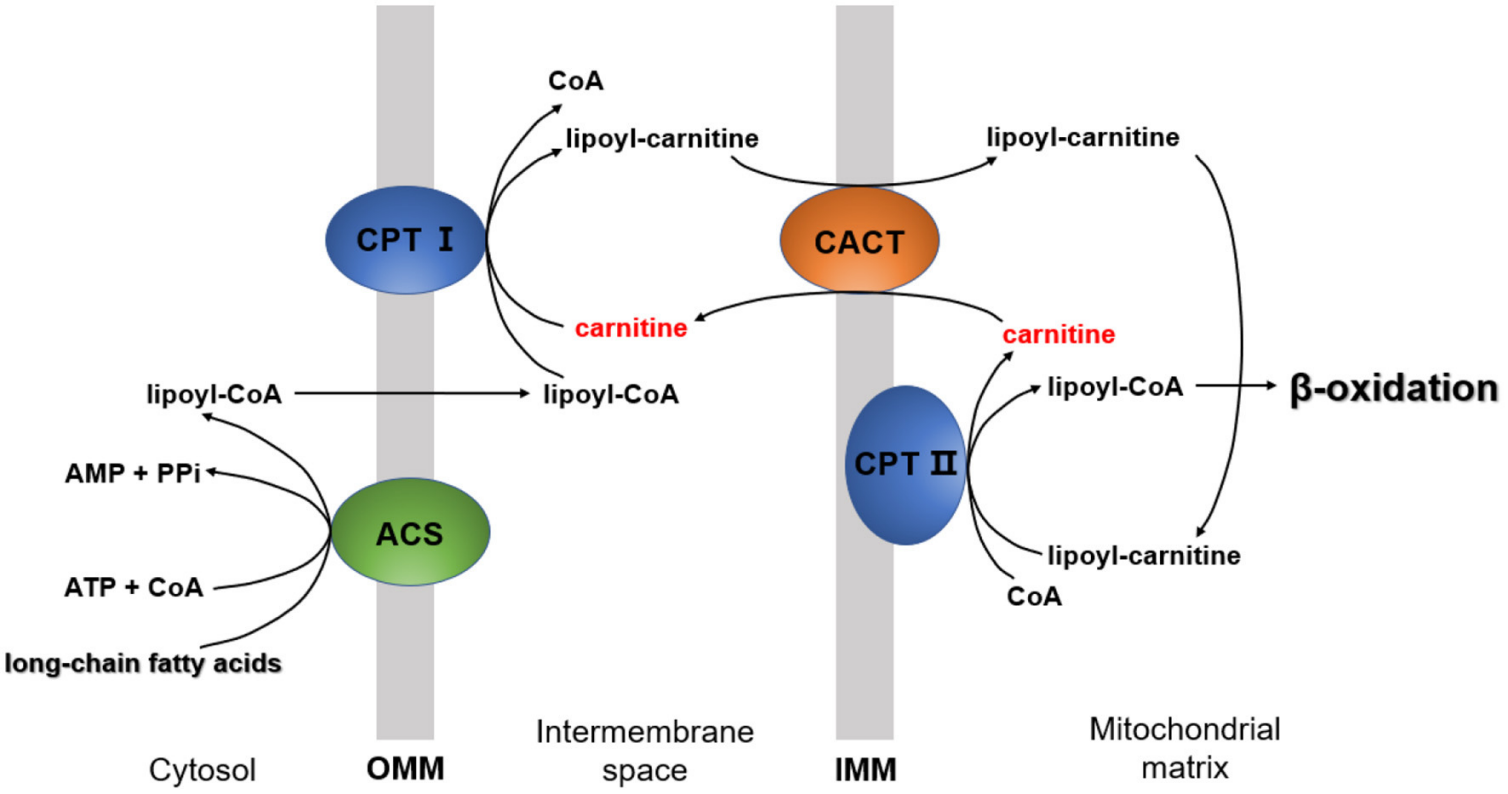
The mechanism by which long-chain fatty acids enter the mitochondria. CPT I, carnitine palmitoyltransferase I; CPT II, carnitine palmitoyltransferase II; ACS, acyl-CoA synthetase; CACT, carnitine-acylcarnitine translocase; OMM, outer mitochondrial membrane; IMM, inner mitochondrial membrane.

The carnitine shuttle has three main steps. First, CoA must be transferred from lipoyl-CoA to the hydroxyl group of carnitine to form lipoyl-carnitine. This transesterification is catalyzed by carnitine palmitoyl transferase I (CPT I) in the outer membrane (1). Second, the lipoyl-carnitine ester enters the matrix by facilitated diffusion through carnitine-acylcarnitine translocase (CACT) located in the inner mitochondrial membrane (31). In the final step, lipoyl-CoA is enzymatically transferred from carnitine to intramitochondrial CoA by carnitine palmitoyl transferase II (CPT II) (32). This isozyme, located on the inner face of the inner mitochondrial membrane, regenerates lipoyl-CoA and releases free carnitine into the matrix. Carnitine enters the intermembrane space again via CACT (Figure 2).

#### Regulation of Acetyl-CoA/CoA Ratio

Under physiological conditions, carnitine can buffer excess acetyl-CoA in the mitochondria via the formation of acetyl-carnitine (33), which requires the presence of carnitine acyltransferase and carnitine acylcarnitine translocase. Acetyl-CoA is either metabolized through the tricarboxylic acid cycle (TCA cycle) or exported as acetyl-carnitine by carnitine. When there is persistent excess or underutilization of certain fatty acids, non-metabolizable acyl-CoAs accumulate. In such situations, carnitine acts as a receiver for these acyl groups by removing them from the tissues and excreting them in the urine (20), or they get separated from carnitine and reused (34). Carnitine regulation of acetyl-CoA/CoA reduces the inhibition of many intramitochondrial enzymes involved in glucose and amino acid catabolism (35).

#### Inter-organellar Acyl Transfer

Long-chain fatty acids (LCFA) and branched chain fatty acids are oxidized in peroxisomes. In contrast to mitochondrial β-oxidation, incomplete peroxisomal oxidation of fatty acids yields acetyl-CoA and shortened medium-chain acyl-CoAs. In order to completely oxidize these substances into CO_2_, the products of peroxisome fatty acid oxidation must be transported to the mitochondria (20). Since CoA and CoA esters cannot penetrate the cell membrane, they must be converted into their respective carnitine esters by catalase and carnitine octyltransferase (COT) in peroxisomes. Therefore, the carnitine esters are transported from peroxisomes to mitochondria through peroxisome and mitochondrial carnitine-acylcarnitine translocase (CACT), then reconverted into CoA esters by mitochondrial CPT II in the mitochondrial matrix (20). These are then oxidized into CO_2_ and H_2_O through mitochondrial β-oxidation, TCA cycle, and electron transfer.

#### Reduction of Oxidative Stress

Carnitine has several protective effects on oxidative stress. These include direct scavenging of free radicals, such as 2,2-diphenyl-1-picrylhydrazyl (DPPH), superoxide dismutase and hydrogen peroxide, and metal chelation to catalyze free radical formation, such as Fe^2+^; inhibition of reactive oxygen species-producing enzymes such as xanthine oxidase (XO) and nicotinamide adenine dinucleotide phosphate oxidase (NOX); upregulation of antioxidant enzymes like catalase (CAT), superoxide dismutase (SOD), glutathione reductase (GR) glutathione peroxidase (GPx), heme oxygenase, endothelial nitric oxide synthase, and other protective proteins (5).

In addition to the above biological functions, carnitine also plays an important role in anti-apoptosis and protection of mitochondrial biogenesis and integrity, which are beyond the scope of this study (5).

### Application in Diseases

Carnitine, a natural compound closely related to the above-mentioned functions, has recently been found to alter the underlying disease pathology with fewer side effects (5, 36). It has been reported that L-carnitine as a supplement can be useful in the treatment of hypertension (37), diabetes mellitus (6, 7), NAFLD (34), heart failure (38), coronary artery disease (39), liver cirrhosis (40), muscle injury (41), dyslipidemia (42), migraine (43), Alzheimer's disease (44), and other chronic diseases.

## Role of Carnitine in the Pathogenesis of NAFLD and Associated Diseases

### Carnitine and “Multiple Hit” Hypothesis in the Pathogenesis of NAFLD

**Figure 3:**
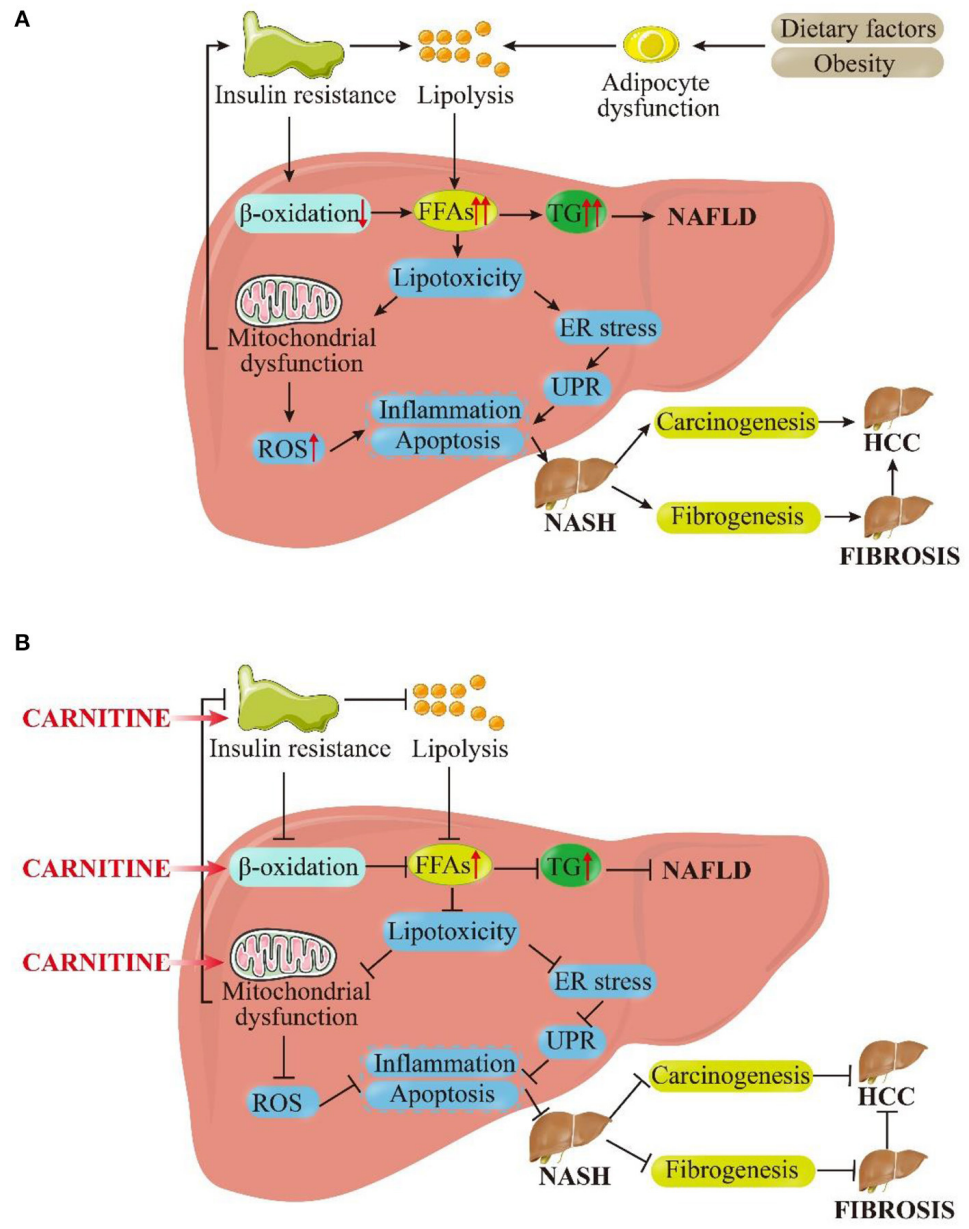
Pictorial representation of the **(A)** pathophysiological mechanism of NAFLD based on the “multiple hit” hypothesis and **(B)** inhibitory effects of carnitine on NAFLD and its progression. As shown in **(A)**, dietary and environmental factors, together with obesity, lead to the proliferation and dysfunction of adipocytes. Insulin resistance acts on adipose tissue and worsens adipocyte dysfunction, which in turn worsens lipolysis. In the liver, insulin resistance inhibits β-oxidation. Under the dual attack of increased lipolysis and weakened β-oxidation, the influx FFAs to hepatocytes is increased greatly, leading to the synthesis and accumulation of TG and enhanced liver lipotoxicity. Excessive TG eventually induces NAFLD. Increased lipotoxicity leads to mitochondrial dysfunction and oxidative stress on endoplasmic reticulum (ER) by the activation of ROS, which leads to liver inflammation and fibrosis. At the same time, mitochondrial dysfunction promotes insulin resistance, exacerbating above process in a vicious circle. These pathological processes can cause the liver to persist in the stable stage of disease (NAFLD) or develop in to NASH. In the late stage of disease progression, NASH can progress to fibrosis or even HCC under the stimulation of certain factors. As shown in **(B)**, carnitine supplementation reduces insulin resistance, promotes β-oxidation and improves mitochondrial function. Subsequently, the cellular concentration of FFAs and TG in hepatocytes get reduced and lipotoxicity is alleviated. The level of ROS is restrained to a certain extent, and inflammation and apoptosis were also improved. These above interlocking effects could alleviate NAFLD and NASH, and they may even have positive therapeutic effects on liver fibrosis and HCC. FFAs, free fatty acids; TG, triglycerides; ER, endoplasmic reticulum; UPR, unfolded protein response; ROS, reactive oxygen species; NASH, non-alcoholic steatohepatitis; HCC, hepatocellular carcinoma.

The prevalence of NAFLD is increasing at a tremendous rate, currently affecting about 24% of the world's population. It has been pinpointed as the most common cause of liver disease globally (45). In the last decade, the clinical burden of NAFLD has been associated to liver-related morbidity and mortality and also to the extra-hepatic manifestations involving other organs and regulatory pathways. The pathogenesis of NAFLD is multifactorial and only partially understood (46). At present, the highly recognized pathogenic mechanism is a “multiple hit” hypothesis, which considers the combined effects of multiple insults on genetically susceptible subjects to induce NAFLD and provides a reasonable explanation for the development of NAFLD (47). The first hit is the accumulation of fat in the liver, followed by the development of necrotic inflammation and fibrosis. In addition, nutritional factors, intestinal microflora, and genetic and epigenetic factors exhibit significant influence (48). Inhibition of β-oxidation, mitochondrial dysfunction, and insulin resistance (IR) are the three most important links in these “hits” as well as targets for carnitine to ameliorate NAFLD. Herein, we attempt to explain the role of carnitine in the pathogenesis of NAFLD, as shown in Figure 3.

Supplementation with carnitine may have a positive effect on the β-oxidation of fatty acids in mitochondria, thereby promoting lipid metabolism by boosting the uptake of fatty acids and eventually reducing fat accumulation in hepatocytes. It is known that NAFLD is associated with an imbalance in a variety of metabolic networks, among which abnormal lipid metabolism is the core pathological metabolic process (49). The immediate cause of NAFLD is high levels of triglycerides (TG) and serum free fatty acids (FFAs). Excess TG comes from hepatic fat production and dietary fat supply, while FFAs accumulate due to lipolysis of visceral adipose tissues (50). The most important strategy to reduce NAFLD is to decrease dietary fat consumption and promote the catabolism of FFAs. β-oxidation is the core process of FFA decomposition, and carnitine acts as an FFA transporter. Carnitine plays an extremely important role in β-oxidation through the “carnitine shuttle,” as described above (51). The carnitine-mediated entry process is a rate-limiting step for mitochondrial LCFA oxidation and, thus, a major regulating point. Increased levels of carnitine may improve the originally inhibited β-oxidation, which is a great way to reduce fat accumulation in the liver (34). Recent studies have emphasized the key role of mitochondrial dysfunction in the occurrence and development of NAFLD (52). The effect of carnitine on mitochondrial dysfunction has been confirmed by various experiments (5). Increased FFAs can lead to hepatic lipotoxicity, generation of reactive oxygen, and damage to the mitochondrial membrane (52). Carnitine treatment could increase the mRNA expression of carnitine palmitoyltransferase 1A and peroxisome proliferator-activated receptor-γ (PPAR-γ), while preventing lipid membrane peroxidation and ROS. This further leads to the reversal of mitochondrial dysfunction, thereby increasing mitochondrial β-oxidation and reducing intracellular oxidative stress to prevent hepatic lipotoxicity (53, 54).

An important mechanism by which carnitine improves IR is enhancing the oxidation of mitochondrial long-chain acyl-CoAs (Figure 2) (55). Accumulation of long-chain acyl-CoAs and other FA metabolites impairs insulin signaling and leads to the development of IR, a crucial pathophysiological factor for carbohydrate metabolism disorders consistent with the development of NAFLD (49). Since abnormal mitochondrial function is associated with IR (56), carnitine therapy can also reduce IR by improving mitochondrial function (56).

Other possible mechanisms to improve insulin sensitivity in NAFLD reported in the literature include the regulation of the acetyl-CoA/CoA ratio in mitochondria, modulation of the pyruvate dehydrogenase complex (PDHC) activity, altering the expression of glycolytic and gluconeogenic enzymes as well as the expression of genes associated with the insulin signaling cascade, and stimulation of the insulin-like growth factor-1 (IGF-1) axis and IGF-1 signaling cascade (34, 55). As mentioned above, carnitine improves the glucose metabolism by reducing acetyl-CoA/CoA and reduces IR. Both insulin and IGF-1 act through their homologous receptor tyrosine kinases to coordinate metabolism and cellular responses to nutrient supply (57). When IGF-1 axis is activated, multiple signaling pathways corresponding to its kinase domain are also activated, including PI3K/Akt pathway and Raf/MEK/ERK level linkage pathway, which can ultimately reduce IR and prevent cell apoptosis (58, 59), thereby improving NAFLD.

### Role of Carnitine in NAFLD Progression and Development of Associated Diseases

The liver is the main organ responsible for detoxification and metabolism of various compounds that produce reactive oxygen species (ROS). As such, liver disease may lead to increased ROS production and, subsequently, increased peroxidation of lipid membranes and inflammatory factors production, leading to hepatocyte injury and cell death (60–63). Injury or death of hepatocytes results in increased release of liver enzymes (64). Patients with chronic liver disease, especially liver cirrhosis, usually suffer from secondary carnitine deficiency (65, 66). Because the liver is one of the main sites for carnitine synthesis, liver disease can impair carnitine synthesis, which further aggravates the above-mentioned pathological processes and promotes disease progression. A recent meta-analysis showed that L-carnitine supplementation improves liver function by reducing histological steatosis and NAS scores in patients with NASH (67). In addition, Okabayashi et al. demonstrated that L-carnitine could improve postoperative liver function in hepatectomized patients (68). L-carnitine reduces inflammatory response via the transfer of β-oxidized long-chain fatty acids in mitochondria and the excretion of toxic substances in fatty acid metabolism (69). It also plays the role of free radical scavenger by reducing the production of ROS (70). Hence, L-carnitine may contribute by reducing oxidative stress, decreasing inflammatory response, and activating enzymes involved in the defense against oxidative damage, thereby reducing elevated serum liver enzymes in patients with liver disease.

Primary carnitine deficiency (PCD) is an autosomal recessive FA oxidation disease (34). Both serum and intracellular carnitine levels are very low in PCD patients. Therefore, FAs cannot be accumulated as an energy source (71), leading to increased ROS production (72). Different from PCD, secondary carnitine deficiency (SCD) is mostly caused by drugs (such as valproic acid, anticancer drugs, omeprazole, amphoteric drugs, etc.) or diseases (such as fatty acid oxidation disorder, organic acidemia, etc.) (1). These drugs or diseases increase the excretion of carnitine in the form of acylcarnitine in urine, reduce renal tubule reabsorption and endogenous biosynthesis, and inhibit carnitine transporters, resulting in increased carnitine consumption (73, 74). Carnitine deficiency impairs the mitochondrial β-oxidation of FAs, leading to acute metabolic decompensation, elevated aminotransferase, and hepatic encephalopathy similar to NAFLD (75). It has been reported that long-term oral carnitine treatment for 6 months in a patient with carnitine deficiency resulted in increased muscle strength, significant reduction in heart size, relief from cardiomyopathy, and partial repletion of carnitine levels in plasma and muscle with complete repletion of liver functions (71). L-carnitine has been approved by the US Food and Drug Administration for the treatment of PCD and SCD (76).

Alcoholic fatty liver disease is another major cause of fatty liver disease, for which carnitine supplementation may also be beneficial for improvement (77). The endogenous biosynthesis of FAs is the main cause of hepatic steatosis in chronic alcoholism (78). Hepatic steatosis is further aggravated by impaired fatty acid oxidation in an ethanol-impaired liver (79). Sachan et al. evaluated the lipid-lowering effects of carnitine and its precursors (lysine and methionine) in rats with chronic alcoholism and concluded that carnitine effectively prevented alcohol-induced hyperlipidemia and liver fat accumulation. Carnitine was also found to be more effective than its precursors (77).

The efficacy of L-carnitine supplements in the treatment of chronic hepatitis B (CHB) remains controversial. A Korean study compared the combination of entecavir and carnitine with entecavir alone to determine their therapeutic effects in CHB. Results indicated that the ALT normalization rate in the combination group was higher than that in the entecavir group, but the HBV-DNA normalization rate and change in serum HBV-DNA levels were similar (80). However, a recent trial showed that besifovir dipivoxil maleate combined with L-carnitine did not lead to any improvement in hepatic steatosis in CHB patients (81). On the other hand, carnitine has a higher recognition for the therapeutic effect in chronic hepatitis C (CHC). A recent meta-analysis showed that long-term use of low-dose (≤2,000 mg/day) L-carnitine could reduce TC and TG in overweight patients with liver disease, especially those with chronic hepatitis C (82). Another study also suggested that L-carnitine may be an effective adjuvant for anti-HCV therapy, because it reduces the oxidative stress induced by JFH-1 infection, inhibits HCV assembly, and exhibits anti-HCV activity through its anti-adipogenic activity in HCV-infected cells (83).

## Downstream Mechanism of Carnitine in NAFLD and Related Diseases

The molecular regulation mechanism of the role of carnitine in fatty acid metabolism has been preliminarily clarified. Typical genes, such as OCTN2 (encoded by SLC22A5) and /or BBD (encoded by BBOX1), regulated by PPARα play regulatory roles in the cellular fatty acid uptake, fatty acid activation, intracellular fatty acid transport, and β-oxidation of mitochondrial and peroxisomal fatty acids (84). With the increase of FFA oxidation, PPARα is activated, and the expression of BBD and OCTN2 in oxidative tissue is simulated to facilitate disposal (85). At the same time, increased expression of BBD and OCTN2 also plays a role in regulating carnitine homeostasis by stimulating the intake and biosynthesis of the carnitine, thus increasing the concentration of carnitine (86). The transcriptional regulation of PPARα on carnitine homeostasis related genes is consistent with their basic role in fatty acid catabolism (85).

Many experimental studies have been conducted to understand the impact of carnitine supplementation in NAFLD. For instance, one study employed a mouse model to demonstrate that L-carnitine supplementation could oppose the NAFLD progression. Results revealed a reduction in hepatic lipid accumulation and oxidative stress, hepatic fibrosis via modulation of α-smooth muscle actin (αSMA), peroxisome-activated receptor gamma (PPARγ), and nuclear factor kappa B (Nf-κB) expression (87). Among them, PPARγ may regulate cell apoptosis through the p21, p53 and p27 pathways, and exert an inhibitory effect on the progression of HCC (88). Another research using a mouse model showed that L-carnitine supplementation can inhibit the NAFLD development by decreasing TNF (receptor superfamily member 9 or CD137), CCL23 (C-C motif chemokine 23), MMP1 (matrix metalloproteinase 1), and FGF21 (fibroblast growth factor 21) in the plasma (89). Another study on hepatoma HepG2 cells revealed the distinct benefits of L-carnitine in fructose-mediated lipid accumulation through adenosine monophosphate-activated protein kinase (AMPK) activation (90). L-carnitine was found to increase PGC1α expression and ameliorate mitochondrial damage (90). Numerous works have revealed that the combination of L-carnitine and nicotinamide nucleoside can enhance the transfer of fatty acids across mitochondrial inner membrane and increase the content of nicotinamide adenine nucleotide (NAD +), which is necessary for β-oxidation and the TCA cycle. It can also reverse the harmful effects of a high-fat diet on liver metabolic pathways and related regulators, such as ACOX, SCAP, SREBF, PPARGC1B, and INSR (91). These mechanisms support the administration of L-carnitine as a novel drug to reduce NAFLD.

## Treatment of NAFLD With L-Carnitine Supplementation

Since the effects of NAFLD are reversible, it is important to control the disease in its early stages (92). Unfortunately, there are no drugs specifically approved for the treatment of NAFLD at present (93). Available treatments for NAFLD mainly focus on changing lifestyle habits and encouraging weight loss. In addition, several molecules have been studied as an adjuvant therapy, including L-carnitine, CoQ_10_, vitamin E, vine tea polyphenol, cytoprotective agents (ursodeoxycholic acid), and insulin sensitizers (pioglitazone and metformin) (94).

L-carnitine, the only biologically active form of carnitine, has been one of the most widely studied molecules for the treatment of NAFLD. Several meta-analyses showed that L-carnitine supplementation improved steatosis and NASH (67) and carnitine supplementation in NAFLD patients could reduce AST, ALT, TG, and HOMA-IR (95). In yet another meta-analysis, L-carnitine supplementation was found to significantly improve the circulating levels of ALT, AST, and gamma-glutamyl transpeptidase (γ-GT), which may have a positive impact on liver function (96).

The research trials conducted to study the effects of L-carnitine supplements in NAFLD, its progression, and the associated diseases are summarized in Tables 1, 2. Most of these studies reported that L-carnitine supplementation can normalize or reduce the serum levels of liver enzymes (94, 97, 98, 101–104). Some have confirmed that L-carnitine supplementation could reduce the incidence and severity of NAFLD (103) and, thus, improve liver attenuation index on CT (101), NAFLD sonographic grade (97), and histological scores (104). Other studies propose that L-carnitine supplementation could improve blood glucose (including FBG and HbA_1_c) and IR (including HOMA-IR) (103), blood lipid profile (104), and mitochondrial function (98, 102) in NAFLD patients with diabetes. Two studies demonstrated that L-carnitine can improve liver function and prognosis in patients with liver cancer after surgery (68, 106). Several randomized controlled clinical trials conducted by Malaguarnera et al. in patients with cirrhosis and hepatic encephalopathy (ranging from mild hepatic encephalopathy to coma) confirmed that L-carnitine supplementation significantly improved hepatic encephalopathy parameters (107–111). The results of these trials suggest that L-carnitine supplementation is advantageous for the treatment of cirrhosis with hepatic encephalopathy. Experimental trials in animals further conclude that L-carnitine supplementation can reduce lipid deposition and increase the metabolites related to β-oxidation, which confirm the importance of L-carnitine in controlling oxidative stress, steatosis, and fibrosis in the liver (87, 99).

**Table 1:** Studies reporting the outcomes of L-carnitine supplementation on NAFLD.

**References**	**Type of study**	**Subject**	**Sample size**	**Duration**	**Intervention group**	**Control group**	Results^*^
Somi et al. (97)	Randomized control trial	Humans	80 (40 vs. 40)	24 weeks	L-Carnitine (250 mg bid)	Without treatment	↓: AST, ALT, BMI, weight, NAFLD sonographic grade
Lim et al. (98)	Randomized control trial	Humans	45 (29 vs. 16)	12 weeks	Carnitine (600 mg/d)	Without treatment	↓: AST, ALT, TB ↑: peripheral mitochondrial DNA copy number
Mollica et al. (87)	Randomized control trial	Mice	30 (10 vs. 10 vs. 10)	6 weeks	Group 1: methionine-choline-deficient diet (6 weeks) + L-carnitine (200 mg/kg each day) (last 3 weeks); Group 2: methionine-choline-deficient diet (6 weeks)	Regular diet (6 weeks)	LC treatment ameliorated hepatic fat accumulation, limited ROS production, improved the fibrosis progression of NAFLD, significantly reduced the NF-kB protein content
Fujisawa et al. (99)	Randomized control trial	Oryzias latipes	80 (20 vs. 20 vs. 20 vs. 20)	6 weeks	Group 1: regular diet (4 weeks) + L-carnitine (1 mM) (2 weeks) Group 3: high-fat diet (4 weeks) + L-carnitine (1 mM) (2 weeks)	Group 2: regular diet (4 weeks) Group 4: high-fat diet (4 weeks)	↓: lipid accumulation ↑: the expression of SOD2, acetyl-CoA, ATP
Kathirvel et al. (100)	Randomized control trial	Mouse	40 (10 vs. 10 vs. 10 vs. 10)	24 weeks	Group 1: high-fat diet Group 2: high-fat diet + ALC + LA	Group 3: standard diet Group 4: standard diet + ALC + LA	↓: size of the mitochondria in liver; ALT; AST ↑: carbamoyl phosphate synthase 1

* *Only show indicators that have statistically significant changes after L-carnitine intervention*.

*ALT, alanine aminotransferase; AST, aspartate aminotransferase; TB, total bilirubin; BMI, body mass index; ROS, reactive oxygen species; SOD2, superoxide dismutase 2*.

**Table 2:** Studies reporting the effects of L-carnitine supplementation on NAFLD progression and related diseases.

**References**	**Type of study**	**Subject**	**Disease**	**Sample size**	**Duration**	**Intervention group**	**Control group**	Results^*^
Alavinejad et al. (94)	Randomized double blind pilot study	Humans	NAFLD + Diabetes	54 (28 vs. 26)	12 weeks	L-Carnitine (750 mg tid)	Placebo (750 mg tid)	↓: AST, ALT
Bae et al. (101)	Randomized double blind pilot study	Humans	NAFLD + Diabetes	78 (39 vs. 39)	12 weeks	Carnitine-orotate complex (824 mg tid)	Placebo (824 mg tid)	↓: ALT, liver attenuation index on CT, HbA_1_c
Hong et al. (102)	Randomized double blind pilot study	Humans	NAFLD +\ Diabetes	52 (26 vs. 26)	12 weeks	Metformin (250 mg tid) + carnitine-orotate complex (300 mg tid)	Metformin (250 mg tid)	↓: ALT, urinary 8-hydroxy-2'-deoxyguanosine ↑: mtDNA copy number
Hamza et al. (103)	Prospective case-control interventional study	Humans	Obesity (suspected NAFLD)	50	24 weeks	Comparison before and after L-Carnitine (50 mg/kg/d) therapy		↓: AST, ALT, Liver span, WC, HC, waist/hip ratio, FBG, Chemerin, HOMA index, incidence and severity of NAFLD (after LC therapy)
Malaguarnera et al. (104)	Randomized double blind pilot study	Humans	NASH	74 (36 vs. 38)	24 weeks	L-carnitine (1 g bid) + 1,600-calorie diet	Placebo (1 g bid) + 1,600-calorie diet	↓: AST, ALT, γ-GT, TC, LDL-C, TG, FBG, HOMA-IR, CRP, TNF-α, histological scores ↑: HDL-C
Ishikawa et al. (105)	Randomized control trial	Mice	NASH	unclear	4 weeks	High-fat diet + L-carnitine	high-fat diet	↓: TNF-α mRNA, NAFLD activity score ↑: CPT II
Okabayashi et al. (68)	Randomized control trial	Humans	liver cancer (after hepatectomy)	208 (102 vs. 106)	2 weeks	L-carnitine (30 mg/kg) (oral before liver resection)	Usual intake	↓: hospital stay, ammonia levels, neutrophil/lymphocyte ratio, post-hepatic liver failure ↑: PT
Hassan et al. (106)	Randomized control trial	Humans	liver cancer (after TACE)	50 (24 vs. 26)	12 weeks	L-Carnitine (300 mg bid)	Usual intake	↓: Child-Pugh score, TB ↑: PT, serum albumin
Malaguarnera et al. (107)	Randomized control trial	Humans	Cirrhosis + HE	120 (60 vs. 60)	60 days	L-Carnitine (2 g bid)	Placebo (2 g bid)	↓: ${\text{NH}}_{4}^{+}$; NCT-A
Cecere et al. (108)	Randomized control trial	Humans	hepatic cirrhosis	27 (16 vs. 11)	4 weeks	L-Carnitine (3 g bid)	Placebo (3g bid)	↓: ${\text{NH}}_{4}^{+}$

* *Only show indicators that have statistically significant changes after L-carnitine intervention*.

*ALT, alanine aminotransferase; AST, aspartate aminotransferase; γ-GT, γ-glutamyl transpeptidase; HDL-C, high dense lipoprotein cholesterol; LDL-C, low density lipoprotein cholesterol; FBG, fasting blood glucose; TB, total bilirubin; TG, triglyceride; TC, cholesterol; WC, waistline circumference; HC, hip circumference; CRP, C-reactive protein; TNF-α, tumor necrosis factor-α; HOMA, homeostatic model assessment; HbA_1_c, glycosylated hemoglobin; TACE, transarterial chemoembolization; PT, prothrombin time; HE, hepatic encephalopathy; NCT-A, number connection test-A*.

At least 11 studies reported no obvious adverse reactions in humans (112–122) and support the use of L-carnitine as an ingredient in dietary supplements. As recognized by several experiments, L-carnitine has also attracted widespread attention since it can be easily obtained from meat at a low-cost.

The recommended dose of L-carnitine is 15 g/day for healthy individuals (123) and 100–400 mg/kg/day for patients with carnitine deficiency (96). Some side effects have been observed after high-dose supplementation of L-carnitine, such as diarrhea, intestinal problems, and trimethylamine production that causes a fishy odor. However, these can be effectively addressed by appropriately reducing the supplemental dosage (124). Hence, it is important to measure the plasma L-carnitine levels to determine the optimal dose of L-carnitine for each patient.

## Limitations and Prospects of Carnitine for the Treatment of NAFLD

Despite promising results, the studies on the role of L-carnitine in NAFLD, listed in Tables 1, 2, face many limitations. First, most trials used the compound preparation of L-carnitine and other substances in the intervention group. The presence of other substances can influence the outcomes of L-carnitine, making it difficult to assess its role in isolation. Second, the bioavailability of L-carnitine was not assessed in the reported studies, so there is no clear reference value for the amount of L-carnitine present in the blood after ingestion. Third, the sample sizes of the reported trials were small, which affects the credibility of the results.

In fact, there are still many unclear aspects of L-carnitine supplementation. For instance, the suggested improvement of metabolism by L-carnitine supplements is based primarily on short-term supplementation (125). A meta-analysis reported that the body weight of obese patients decreased significantly after supplementation with L-carnitine (126). However, in the subgroup analysis, it was found that L-carnitine had no effect on the body weight of subjects whose BMI was <25 kg/m^2^ or when L-carnitine supplementation was administered for more than 24 weeks (126). Thus, it is not explicit whether the effects of L-carnitine supplementation on NAFLD will result in similar time-dependent effects. Although carnitine supplementation was given orally in most of the studies (Tables 1, 2), some trials administered carnitine intravenously (127, 128). Therefore, the optimal route to administer L-carnitine supplements for NAFLD remains unclear. While many substances can be used as dietary supplements to improve NAFLD, no studies have directly compared them to determine which is most effective. Furthermore, the studies on the effects of L-carnitine supplements in NAFLD have yielded conflicting results. Bruls et al. showed that L-carnitine infusion could neither alleviate lipid-induced insulin resistance and metabolic inflexibility nor change the availability of skeletal muscle carnitine (129). Fujiwara et al. found that high-fat diet feeding and L-carnitine supplementation increased STAT3 phosphorylation in HCC tissues and could synergistically promote the development of liver cancer (130).

To improve the accuracy of future studies on the effects of L-carnitine supplementation in NAFLD treatment, the following aspects could be considered: use of L-carnitine in isolation in the intervention group; assessment of the bioavailability of L-carnitine; multicenter trials with larger sample size; longer follow-up to evaluate time-dependent effects; and use of intravenous L-carnitine supplementation. In addition, experimental studies are recommended to compare the effects of other substances that have been proven effective in enhancing NAFLD via L-carnitine and to understand the downstream mechanism.

## Conclusion

Carnitine plays an important role in transporting FAs to the mitochondrial matrix for β-oxidation, regulating acetyl-CoA/CoA, exporting acetyl- and chain-shortened acyl products from peroxisomes, and reducing oxidative stress. L-carnitine supplementation can normalize or reduce serum levels of liver enzymes, decrease the incidence and severity of NAFLD, and improve both the lipid profile and mitochondrial function. L-carnitine may have therapeutic effects on liver diseases, including NASH, cirrhosis, HCC, alcoholic fatty liver disease, and viral hepatitis. In addition, L-carnitine supplementation is safe, low cost, and easy to administer. Due to the limited and inadequate studies on the effects of L-carnitine supplementation on NAFLD, future research should aim to determine the exact role of L-carnitine for the treatment of NAFLD.

## Author Contributions

NL: study design and drafting. HZ: study design and critical review/revision. All authors contributed to the article and approved the submitted version.

## Conflict of Interest

The authors declare that the research was conducted in the absence of any commercial or financial relationships that could be construed as a potential conflict of interest.

## Publisher's Note

All claims expressed in this article are solely those of the authors and do not necessarily represent those of their affiliated organizations, or those of the publisher, the editors and the reviewers. Any product that may be evaluated in this article, or claim that may be made by its manufacturer, is not guaranteed or endorsed by the publisher.

## Abbreviations

ACS: acyl-CoA synthetase
ALT: alanine aminotransferase
AMPK: adenosine monophosphate-activated protein kinase
AST: aspartate aminotransferase
BMI: body mass index
CACT: carnitine-acylcarnitine translocase
CAT: catalase
CHB: chronic hepatitis B
CHC: chronic hepatitis C
COT: carnitine octyltransferase
CPT I: carnitine palmitoyltransferase I
CPT II: carnitine palmitoyltransferase II
CRP: C-reactive protein
ER: endoplasmic reticulum
FBG: fasting blood glucose
FFAs: free fatty acids
GPx: glutathione peroxidase
GR: glutathione reductase
HbA1c: glycosylated hemoglobin
HC: hip circumference
HCC: hepatocellular carcinoma
HDL-C: high dense lipoprotein cholesterol
HE: hepatic encephalopathy
HOMA: homeostatic model assessment
IGF-1: insulin-like growth factor-1
IMM: inner mitochondrial membrane
IR: insulin resistance
LCFA: long-chain fatty acids
LDL-C: low density lipoprotein cholesterol
NAFLD: non-alcoholic fatty liver disease
NASH: non-alcoholic steatohepatitis
NCT-A: number connection test-ANf-κB, nuclear factor kappa B
NOX: nicotinamide adenine dinucleotide phosphate oxidase
OMM: outer mitochondrial membrane
PCD: primary carnitine deficiency
PDHC: pyruvate dehydrogenase complex
PPARγ: peroxisome-activated receptor-γ
PT: prothrombin time
ROS: reactive oxygen species
SOD: superoxide dismutase
SOD2: superoxide dismutase 2
TACE: transarterial chemoembolization
TB: total bilirubin
TC: cholesterol
TG: triglyceride
TNF-α: tumor necrosis factor-α
UPR: unfolded protein response
WC: waistline circumference
XO: xanthine oxidase
αSMA: α-smooth muscle actin
γ-GT: γ-glutamyl transpeptidase.
